# Clinical and electrocardiographic predictors of perfusion impairment in symptomatic bradycardia requiring permanent pacemaker implantation

**DOI:** 10.17305/bb.2026.14123

**Published:** 2026-05-05

**Authors:** Rauf Avcı, Muhammed Rıdvan Ersoysal, Görkem Kuş, Sever Yılmaz, Göksel Çağırcı, Şakir Arslan, Akif Durak, Ayşegül Ülgen Kunak, Ayşe Karaduru Avcı

**Affiliations:** 1 Turkish Ministry of Health Antalya Training and Research Hospital, Antalya, Türkiye; 2 Department of Internal Medicine, Antalya City Hospital, Kepez, Antalya, Türkiye

**Keywords:** Bradycardia, atrioventricular block, pacemaker, hemodynamics, risk factors

## Abstract

Symptomatic bradycardia can lead to systemic hypoperfusion and hemodynamic instability, particularly in patients with advanced conduction system disease. This study aimed to identify factors associated with perfusion impairment in patients experiencing persistent symptomatic bradycardia who required permanent pacemaker implantation. In this single-center retrospective observational study, 299 patients who underwent pacemaker implantation between December 2021 and June 2025 were screened, resulting in the inclusion of 166 patients with persistent symptomatic bradycardia. Patients were stratified based on the presence of perfusion impairment, defined using specific clinical, biochemical, and organ-specific criteria. We analyzed clinical, laboratory, electrocardiographic, and echocardiographic parameters obtained prior to pacing. Perfusion impairment was identified in 70 patients, who were older and exhibited lower heart rates and blood pressures, elevated lactate levels, a higher incidence of renal dysfunction, and increased inflammatory markers. Multivariable analysis revealed that age (odds ratio [OR] 1.097; *P* ═ 0.001), heart rate (OR 0.910; *P* ═ 0.022), and high-grade atrioventricular (AV) block (OR 7.546; *P* ═ 0.002) were independently associated with perfusion impairment. Receiver operating characteristic (ROC) analysis demonstrated moderate discrimination for age and heart rate, while the multivariable model displayed improved discrimination. Our findings indicate that advanced age, lower baseline heart rate, and high-grade AV block are correlated with perfusion impairment in patients with persistent symptomatic bradycardia requiring pacemaker implantation. Early identification of this high-risk subgroup may facilitate timely monitoring and intervention.

## Introduction

Bradycardia is defined as a heart rate of fewer than 60 beats per minute [1]. While it may occur physiologically in athletes or during sleep, bradycardia can also develop as part of the normal aging process [2]. Symptomatic bradycardia is characterized as documented bradyarrhythmia that results in clinical manifestations, such as syncope, presyncope, transient dizziness, lightheadedness, heart failure symptoms, or confusion, due to reduced cerebral perfusion caused by the slow heart rate [1–3].

Although some individuals may remain entirely asymptomatic, bradycardia can lead to serious, potentially life-threatening clinical conditions in others [4]. The development of these clinical presentations is influenced by the duration of bradycardia (persistent or intermittent), its severity, and the resulting reduction in cardiac output [5]. Underlying causes range widely from sinus node dysfunction to atrioventricular (AV) conduction block. Management strategies vary according to etiology, including options ranging from conservative monitoring to permanent pacemaker implantation [6].

AV conduction disturbances are classified into first-, second-, and third-degree blocks. Although first-degree AV block is generally benign, second- and third-degree AV blocks may lead to hemodynamic instability and impaired perfusion [6]. In the acute management of patients exhibiting hemodynamic compromise, treatment may involve pharmacologic therapy or temporary pacing, depending on the underlying cause [1]. Patients diagnosed with bradycardia often remain under observation in emergency departments or intensive care units while awaiting permanent pacemaker implantation, and perfusion impairment may occur during this period.

While previous studies have extensively examined factors contributing to the development of bradycardia [7], data regarding the patient groups prone to hemodynamic and perfusion disturbances remain limited. Early identification of high-risk patients is essential for preventing potential complications. Therefore, this study aimed to investigate factors associated with the development of perfusion impairment in patients presenting to the emergency department with symptomatic bradycardia.

## Materials and methods

### Study design and population

This retrospective observational study included patients who underwent permanent pacemaker implantation for symptomatic bradycardia between December 2021 and June 2025 at Antalya Training and Research Hospital. The study protocol received approval from the Antalya Training and Research Hospital Clinical Research Ethics Committee (Approval No: 12/16; July 17, 2025). Clinical data were obtained from the hospital’s electronic medical record system, archived reports, outpatient clinic notes, and patient files.

**Figure 1.**
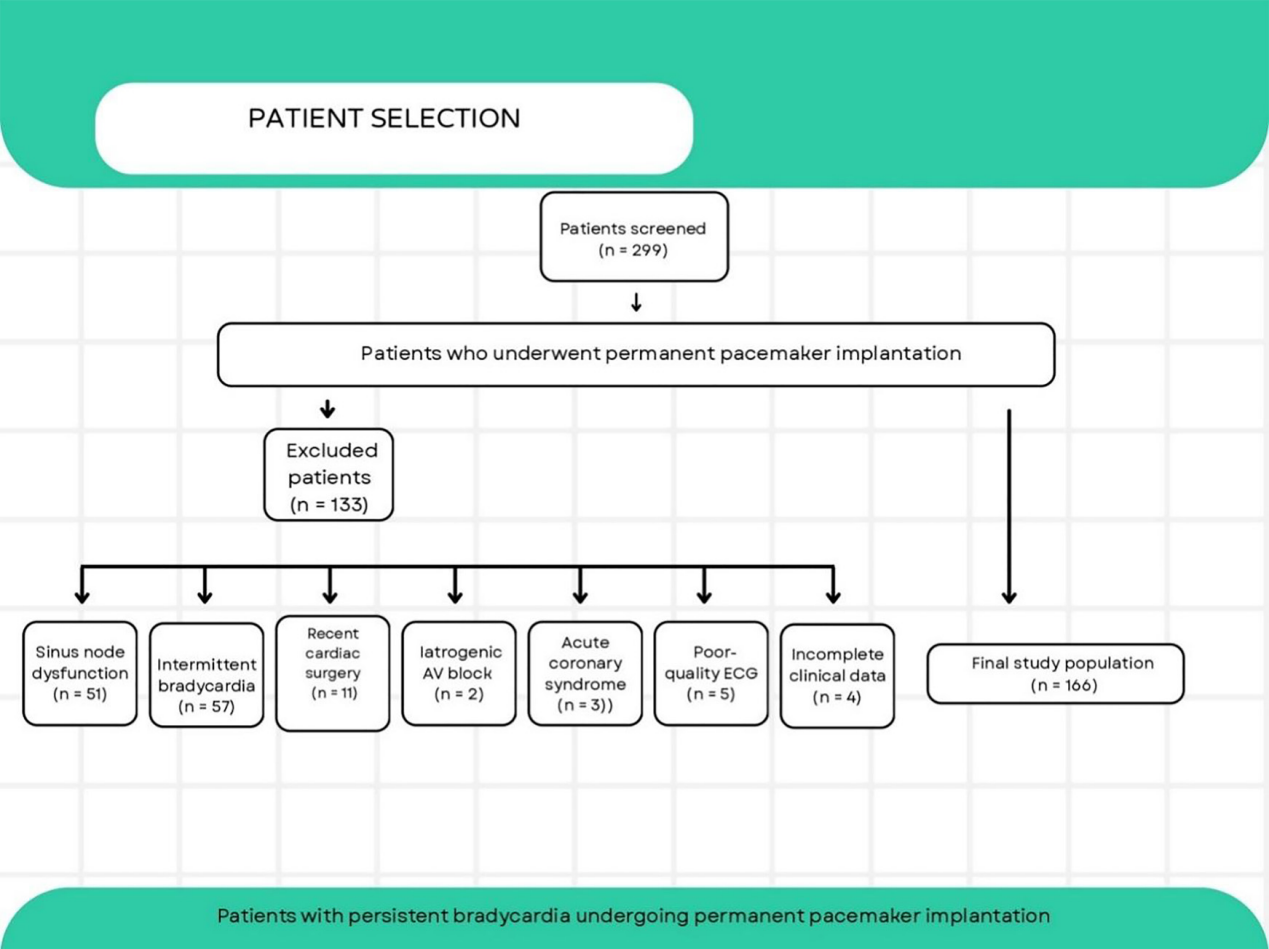
**Flowchart of patient selection**.

A total of 299 patients were initially screened. As this study focused specifically on patients requiring permanent pacemaker implantation for persistent symptomatic bradycardia, exclusion criteria were structured into two categories to ensure a homogeneous study population. First, clinical exclusions were applied to patients with sinus node dysfunction, intermittent bradycardia that did not persist until the time of implantation, and other rhythm disturbances not meeting the study definition of intrinsic conduction system disease. Second, data-quality exclusions were performed for patients with poor-quality electrocardiogram (ECG) recordings or incomplete clinical records. Consequently, 133 patients were excluded, and the final analysis was conducted with 166 patients. The patient selection process is detailed in the flow diagram (Figure 1).

Patients aged ≥18 years who demonstrated persistent bradycardia on electrocardiography (ECG) from the time of hospital admission until pacemaker implantation were included. Eligible rhythm disorders included low-ventricular-rate atrial fibrillation, nodal rhythm, 2:1 AV block, high-grade AV block, and complete AV block. Bradyarrhythmias were defined according to contemporary guideline recommendations [6].

In this study, electrocardiographic conduction abnormalities were categorized into distinct groups to avoid overlap. Specifically, 2:1 AV block, complete AV block, and high-grade AV block were analyzed as separate entities. High-grade AV block was defined as advanced AV conduction disturbances characterized by three or more consecutive non-conducted *P* waves. The heart rate used in the analyses was defined as the baseline value obtained from the ECG at presentation prior to pacing. All ECG and laboratory parameters were obtained at presentation before pacing. Patients with incomplete clinical data were excluded from the analysis. Endpoint adjudication was performed through retrospective chart review by two independent investigators using predefined criteria, with discrepancies resolved by consensus.

Perfusion impairment was defined according to prespecified clinical, laboratory, and organ-specific parameters (Table 1) [8]. A diagnosis of perfusion impairment was based on the presence of at least one predefined objective clinical or biochemical abnormality. Improvement after temporary or permanent pacing was considered supportive evidence for bradycardia-related hypoperfusion but was not required for diagnosis.

**Table 1:** Criteria for perfusion impairment

**Clinical Parameter**	**Definition/criterion**	**Explanation**
Hypotension	Systolic blood pressure <90 mmHg	Clinical indicator of impaired systemic perfusion
Elevated lactate	Serum lactate ≥ 2 mmol/L	Biochemical marker of hypoperfusion
AKI	Increase in serum creatinine ≥0.3 mg/dL within 48 h, or ≥1.5× baseline within 7 days	Defined according to KDIGO 2012 criteria [8]
Hepatic injury	ALT and/or AST >2× upper limit of normal compared with baseline values within 3 months	Reflects hepatic hypoperfusion
Altered mental status	New-onset confusion, somnolence, or decreased consciousness level	Attributed to cerebral hypoperfusion

Improvement following temporary or permanent pacing was regarded as supportive evidence for bradycardia-related hypoperfusion, although it was not a prerequisite for diagnosis. Abbreviations: AKI, acute kidney injury; ALT, alanine aminotransferase; AST, aspartate aminotransferase; KDIGO, Kidney Disease: Improving Global Outcomes.

At presentation, all patients underwent laboratory testing, a 12-lead ECG, and transthoracic echocardiographic evaluation, performed according to standard imaging protocols. ECGs were independently reviewed by two cardiologists, and heart rate and QRS duration were obtained from automated device measurements.

### Statistical analysis

All statistical analyses were performed using IBM SPSS Statistics for Windows, Version 27.0 (IBM Corp., Armonk, NY, USA). Continuous variables were expressed as mean ± standard deviation or median (interquartile range), and categorical variables as frequencies and percentages. Group comparisons between patients with and without perfusion impairment were conducted using the Student’s *t*-test or Mann–Whitney *U* test for continuous variables and the chi-square test or Fisher’s exact test for categorical variables, as appropriate.

To evaluate factors associated with perfusion impairment, univariate logistic regression analyses were initially performed for all candidate variables. Variables for the multivariable logistic regression model were selected based on a combination of univariable analysis (*P* < 0.10) and clinical relevance. The candidate set included age, heart rate, high-degree AV block, chronic kidney disease (CKD), C-reactive protein (CRP), albumin, and troponin status. The multivariable model was constructed using the enter method, with all selected variables included simultaneously. Multicollinearity was assessed prior to model construction. The linearity assumption of continuous variables (age and heart rate) on the log-odds scale was assessed using the Box–Tidwell test, and no significant deviation from linearity was observed (*P* ═ 0.563 for age and *P* ═ 0.305 for heart rate). Model calibration was assessed using the Hosmer–Lemeshow goodness-of-fit test. Calibration was further evaluated using the calibration slope, calculated by regressing the observed outcome on the logit of predicted probabilities.

Odds ratios (ORs) and 95% confidence intervals (CIs) were reported for each variable.

To address potential confounding, a 1:1 propensity score matching (PSM) analysis was performed as a sensitivity analysis using a nearest-neighbor algorithm with a caliper width of 0.2. Patients with and without perfusion impairment were matched based on age, heart rate, systolic blood pressure, and creatinine levels. Covariate balance after matching was assessed using standardized mean differences (SMDs) to ensure comparability between groups. An SMD value of less than 0.10 was considered to indicate excellent balance between the two groups.

To explore the potential clinical applicability of the identified variables, a simplified exploratory risk score was constructed as a secondary analysis based on the multivariable logistic regression model. Variables were dichotomized using receiver operating characteristic (ROC)-derived cut-off values and weighted according to their regression coefficients. The total score ranged from 0–5 points. The exploratory risk score was categorized into three levels: low, intermediate, and high risk. The association between these risk categories and the prevalence of perfusion impairment was assessed using the Pearson chi-Square test.

ROC curve analyses were performed to determine the discriminative ability of age and heart rate in identifying perfusion impairment. The area under the curve (AUC), optimal cut-off values, sensitivity, and specificity were calculated using the Youden index. Internal validation was performed using a bootstra*p* resampling procedure with 1000 iterations to assess the stability of the model estimates and the robustness of the model findings.

A *P*-value < 0.05 was considered statistically significant. Missing data were minimal (<5% for all variables), and a complete-case analysis approach was applied. Given the low proportion of missing data, no imputation methods were considered necessary.

**Table 2:** Comparative analysis of clinical, laboratory, echocardiographic, and electrocardiographic parameters in patients with and without perfusion impairment

**Variable**	**No perfusion impairment (*n* ═ 96)**	**Perfusion impairment (*n* ═ 70)**	***P* value**
SBP (mmHg)	151.90 ± 31.79	132.73 ± 32.47	<0.001
DBP (mmHg)	73.66 ± 17.31	67.96 ± 16.19	0.033
Lactate (mmol/L)	1.28 ± 0.44	2.51 ± 1.12	<0.001
Age (years)	72.67 ± 8.87	79.24 ± 8.23	<0.001
WBC (×10^3^/µL)	8.07 ± 2.34	9.79 ± 3.30	<0.001
Neutrophils (×10^3^/µL)	5.20 ± 2.05	7.04 ± 3.21	<0.001
Lymphocytes (×10^3^/µL)	2 (1.5–2.6)	1.7 (1.2–2.3)	0.046
Hemoglobin (g/dL)	12.7 ± 1.69	12.01 ± 1.88	0.014
Platelets (×10^3^/µL)	221.4 ± 76.8	213.4 ± 104.1	0.568
BUN (mg/dL)	23.22 ± 10.55	32.56 ± 15.52	<0.001
Creatinine (mg/dL)	1.20 ± 0.52	1.48 ± 0.53	0.001
eGFR (mL/min/1.73 m^2^)	60.92 ± 19.11	44.86 ± 18.92	<0.001
Sodium (mmol/L)	139.28 ± 2.83	138.17 ± 4.53	0.074
Potassium (mmol/L)	4.38 ± 0.43	4.59 ± 0.62	0.010
Calcium (mg/dL)	9.08 ± 0.64	9.12 ± 0.72	0.687
Magnesium (mg/dL)	2.02 ± 0.30	2.04 ± 0.33	0.732
Albumin (g/L)	38.30 ± 3.90	36.41 ± 4.04	0.003
Troponin (ng/L)	12 (5–26)	31 (14–61)	<0.001
CR*P* (mg/L)	4.6 (1.7–8.6)	7.3 (2.4–18.6)	0.009
LVDD (mm)	47.95 ± 4.58	46.13 ± 4.11	0.012
LVSD (mm)	30.40 ± 5.33	29.45 ± 4.19	0.241
IVSd (mm)	11.90 ± 1.95	12.18 ± 1.65	0.344
LVPWT (mm)	11.27 ± 1.56	11.86 ± 1.56	0.020
Left atrial diameter (mm)	39.78 ± 6.33	39.99 ± 5.50	0.833
sPA*P* (mmHg)	37.96 ± 12.90	38.85 ± 15.59	0.722
Heart rate (bpm)	39.65 ± 5.25	34.07 ± 6.65	<0.001
QRS duration (ms)	114.43 ± 45.78	122.25 ± 26.01	0.209
QTc (ms)	464.73 ± 53.33	497.57 ± 52.50	0.012
Rate-limiting drugs	25 (26.0%)	20 (28.6%)	0.727
Female sex	45 (46.9%)	35 (50%)	0.754
Hypertension	62 (64.6%)	51 (73.9%)	0.236
Diabetes mellitus	38 (39.6%)	25 (36.2%)	0.746
Coronary artery disease	23 (24.0%)	18 (25.8%)	0.402
Chronic kidney disease	26 (27.1%)	34 (48.6%)	0.005
LVEF 40–50%	6 (6.3%)	3 (4.3%)	0.489
LVEF <40%	1 (1.0%)	0 (0.0%)	NA
Troponin (+)	23 (24.0%)	31 (44.3%)	0.017
Moderate/severe valvular disease	59 (61.5%)	44 (62.9%)	0.546
Complete AV block	22 (22.9%)	21 (30.0%)	0.370
High-grade AV block	7 (7.3%)	32 (45.7%)	<0.001
2:1 AV block	47 (49.0%)	5 (7.1%)	<0.001
Nodal rhythm	11 (11.5%)	7 (10.0%)	0.806
Atrial fibrillation with slow ventricular response	9 (9.4%)	5 (7.1%)	0.779

Continuous variables are reported as the mean ± SD for normally distributed data and as the median (IQR) for non-normally distributed data. Group comparisons were conducted using either Student’s *t*-test or the Mann–Whitney *U* test, depending on the data distribution. Abbreviations: AV, atrioventricular; BUN, blood urea nitrogen; CRP, C-reactive protein; DBP, diastolic blood pressure; eGFR, estimated glomerular filtration rate; IQR, interquartile range; IVSd, interventricular septal thickness in diastole; LVEF, left ventricular ejection fraction; LVDD, left ventricular diastolic diameter; LVPWT, left ventricular posterior wall thickness; LVSD, left ventricular systolic diameter; NA, not applicable; QRS, QRS complex; QTc, corrected QT interval; SBP, systolic blood pressure; SD, standard deviation; sPAP, systolic pulmonary artery pressure; WBC, white blood cell.

## Results

A total of 166 patients were included in this study. Based on predefined criteria, 70 patients exhibited perfusion impairment, whereas 96 patients did not. Patients were stratified into two groups according to the presence or absence of perfusion impairment. A comprehensive comparison of clinical, laboratory, echocardiographic, and electrocardiographic characteristics is presented in Table 2.

Patients with perfusion impairment were significantly older (79.2 ± 8.2 vs. 72.7 ± 8.9 years, *P* < 0.001) and exhibited lower systolic and diastolic blood pressure values (both *P* < 0.05). Markers of tissue hypoperfusion, particularly lactate levels, were markedly elevated in this group (*P* < 0.001). Inflammatory markers, including white blood cell (WBC) and neutrophil counts, were higher, whereas hemoglobin and albumin levels were lower (all *P* < 0.05). Renal dysfunction was more prevalent among patients with perfusion impairment, reflected by significantly higher blood urea nitrogen (BUN) and creatinine levels and reduced estimated glomerular filtration rate (eGFR) (all *P* < 0.001). Electrolytes were overall comparable, except for potassium, which was higher in the perfusion impairment group (*P* ═ 0.010).

**Table 3:** Distribution of components contributing to perfusion impairment

**Component of Perfusion İmpairment**	***n* (%)**
Hypotension (SB *P* < 90 mmHg)	6 (8.6%)
Elevated lactate (≥2 mmol/L)	37 (52.9%)
Acute kidney injury	33 (47.1%)
Hepatic injury	2 (2.9%)
Altered mental status	9 (12.9%)

The components of perfusion impairment were not mutually exclusive: Individual patients could fulfill multiple criteria; Percentages were calculated based on the 70 patients exhibiting perfusion impairment. Abbreviations: SBP, systolic blood pressure.

**Figure 2.**
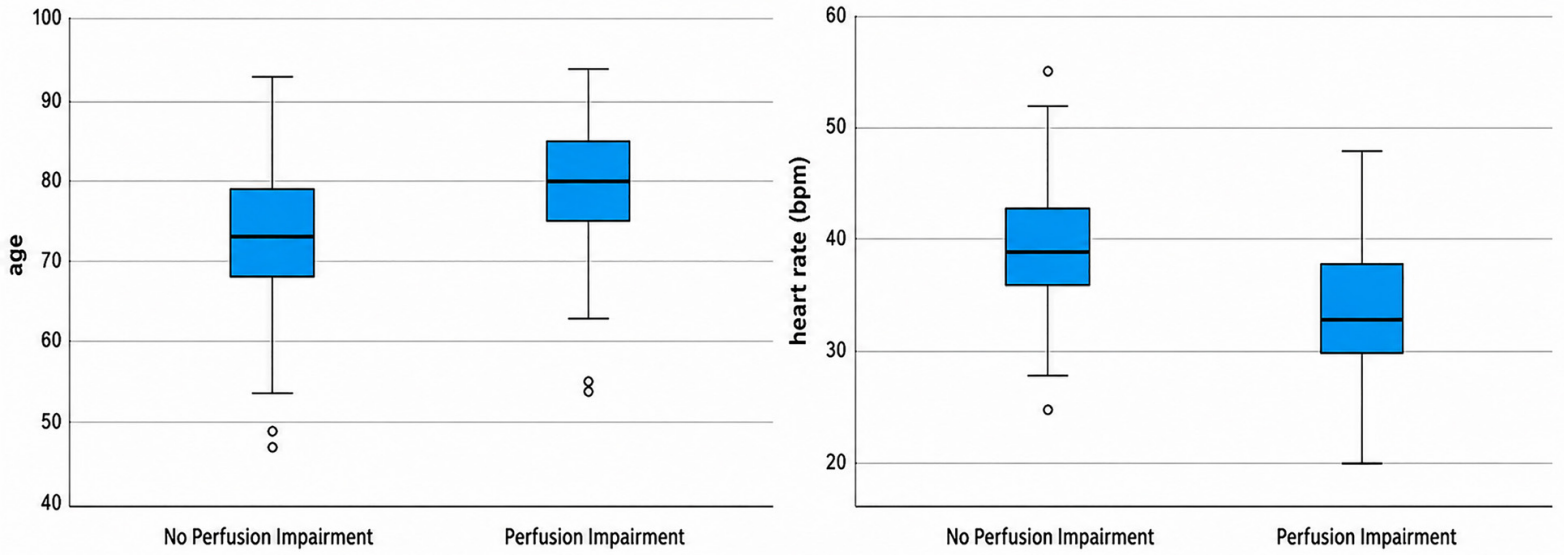
**Age and baseline heart rate according to perfusion impairment status.** Boxplots show the distribution of age and baseline heart rate in patients with and without perfusion impairment. Patients with perfusion impairment were significantly older than those without perfusion impairment and had markedly lower baseline heart rates at presentation (both *P* < 0.001). The central line represents the median, boxes indicate the interquartile range, whiskers show the data range excluding outliers, and circles represent outlier values. Abbreviations: Bpm, beats per minute; IQR, interquartile range.

**Figure 3.**
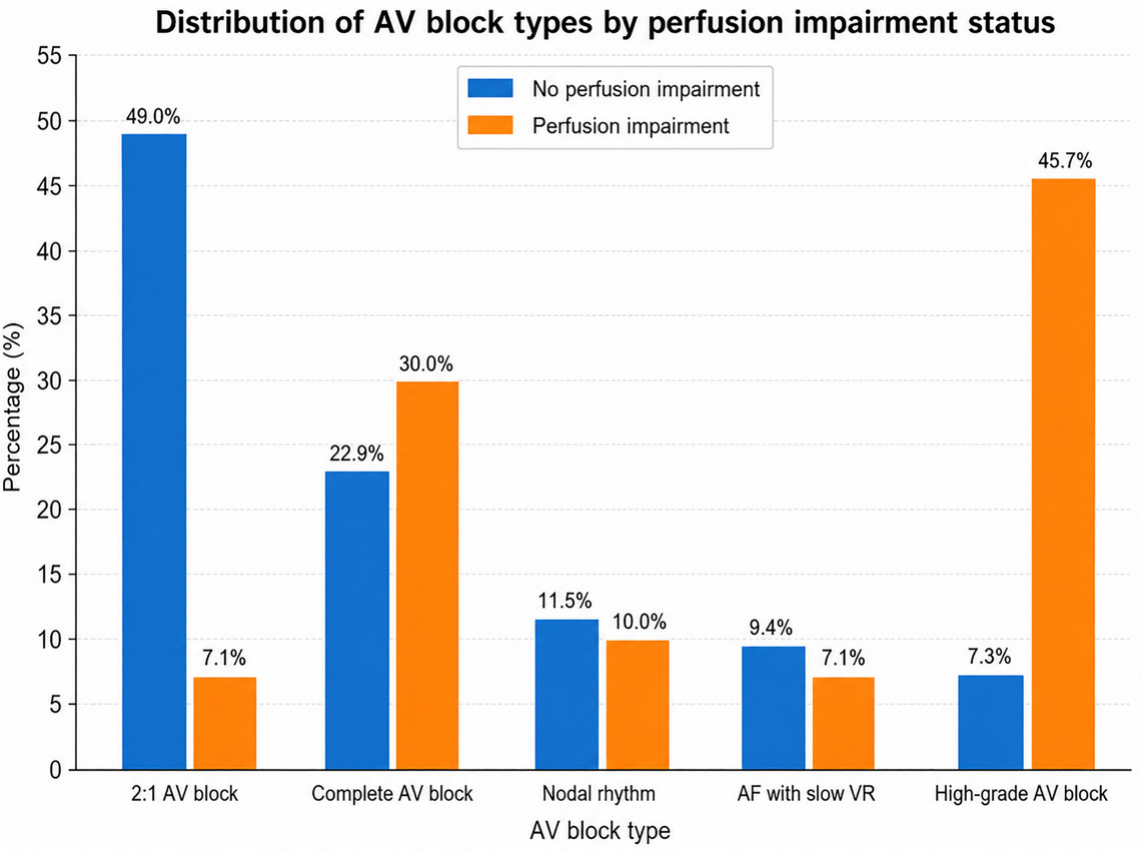
**Distribution of bradyarrhythmia types according to perfusion impairment status.** Bar chart shows the distribution of baseline bradyarrhythmia types in patients without perfusion impairment (upper panel) and with perfusion impairment (lower panel). The prevalence of conduction abnormalities differed markedly between groups. High-grade atrioventricular block was substantially more frequent among patients with perfusion impairment than among those without perfusion impairment (45.7% vs. 7.3%), whereas 2:1 atrioventricular block was markedly less common (7.1% vs. 49.0%). Complete atrioventricular block was also more frequent in the perfusion impairment group (30.0% vs. 22.9%), while nodal rhythm (10.0% vs. 11.5%) and atrial fibrillation with slow ventricular response (7.1% vs. 9.4%) occurred at comparable frequencies. Abbreviations: AF, atrial fibrillation; AV, atrioventricular; VR, ventricular response.

Electrocardiographically, patients with perfusion impairment demonstrated significantly lower heart rates (34.1 ± 6.7 vs. 39.7 ± 5.3 bpm, *P* < 0.001) and longer QTc intervals (*P* ═ 0.012). The distribution of age and heart rate according to perfusion status is illustrated in Figure 2. The distribution of AV conduction abnormalities differed markedly between groups (Figure 3). High-grade AV block was substantially more frequent in the perfusion impairment group (45.7% vs. 7.3%, *P* < 0.001), whereas 2:1 AV block was significantly less common (*P* < 0.001).

The distribution of individual components of perfusion impairment is illustrated in Table 3. Elevated lactate levels (52.9%) and acute kidney injury (47.1%) were the most frequent components, whereas hypotension (8.6%) and hepatic injury (2.9%) were less common.

**Figure 4.**
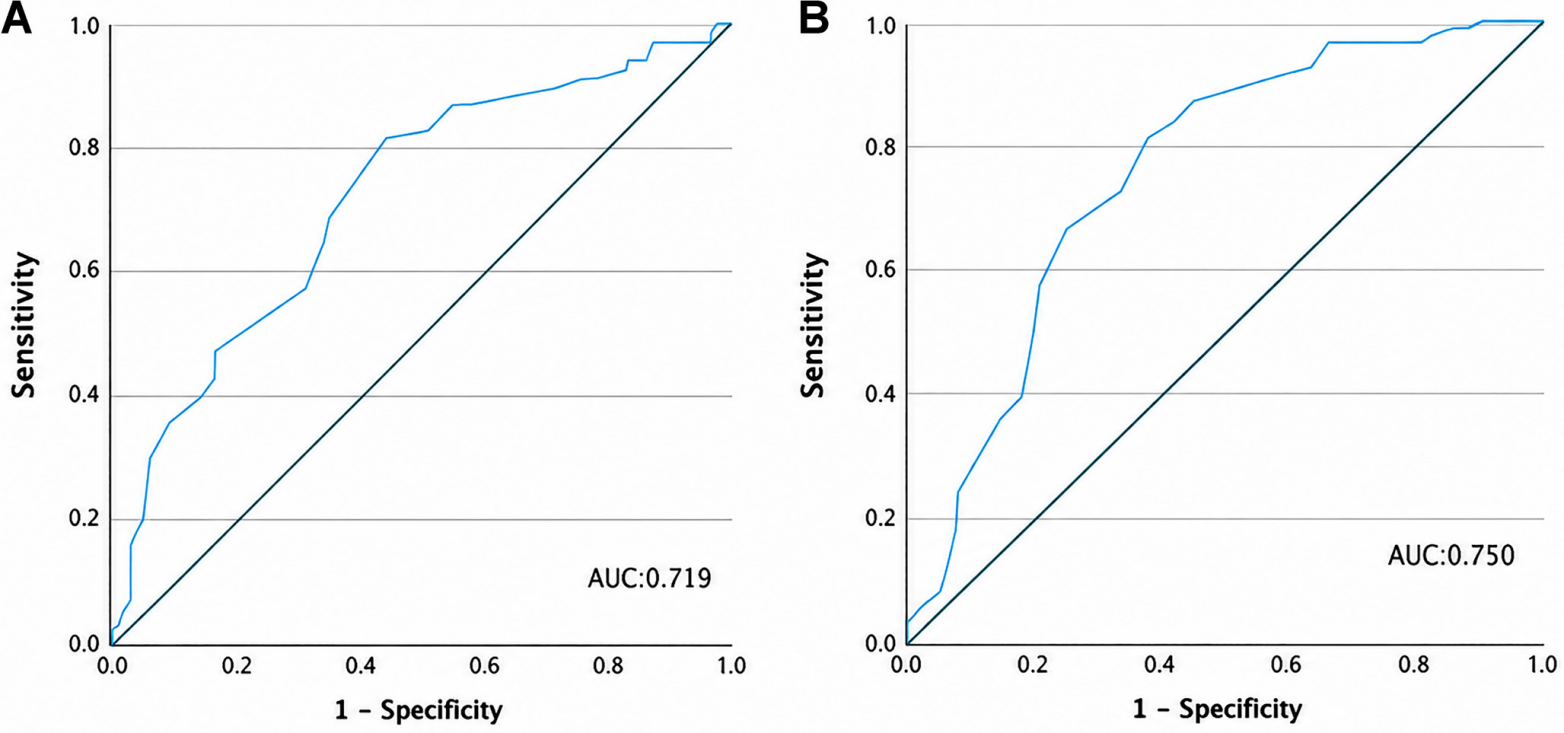
**ROC curves of age and baseline heart rate for identifying perfusion impairment.** (A) ROC curve for age, showing moderate discriminative ability for identifying perfusion impairment, with an area under the curve of 0.719. The optimal cut-off value determined by the Youden index was ≥72.5 years, corresponding to a sensitivity of 82.9% and a specificity of 49.0%. (B) ROC curve for baseline heart rate, also demonstrating moderate discriminative ability, with an area under the curve of 0.750. The optimal cut-off value was ≤33.5 beats per minute, with a sensitivity of 87.5% and a specificity of 54.3%. The diagonal reference line indicates no discriminative ability. Abbreviations: AUC, area under the curve; bpm, beats per minute; ROC, receiver operating characteristic.

ROC analyses of individual variables (age and heart rate) are presented as secondary analyses (Figure 4, Table 4). Both parameters demonstrated moderate discriminative ability. Age yielded an AUC of 0.719, with an optimal cut-off of ≥72.5 years (sensitivity 82.9%, specificity 49.0%), while heart rate yielded an AUC of 0.750, with a cut-off of ≤33.5 bpm (sensitivity 87.5%, specificity 54.3%).

**Figure 5.**
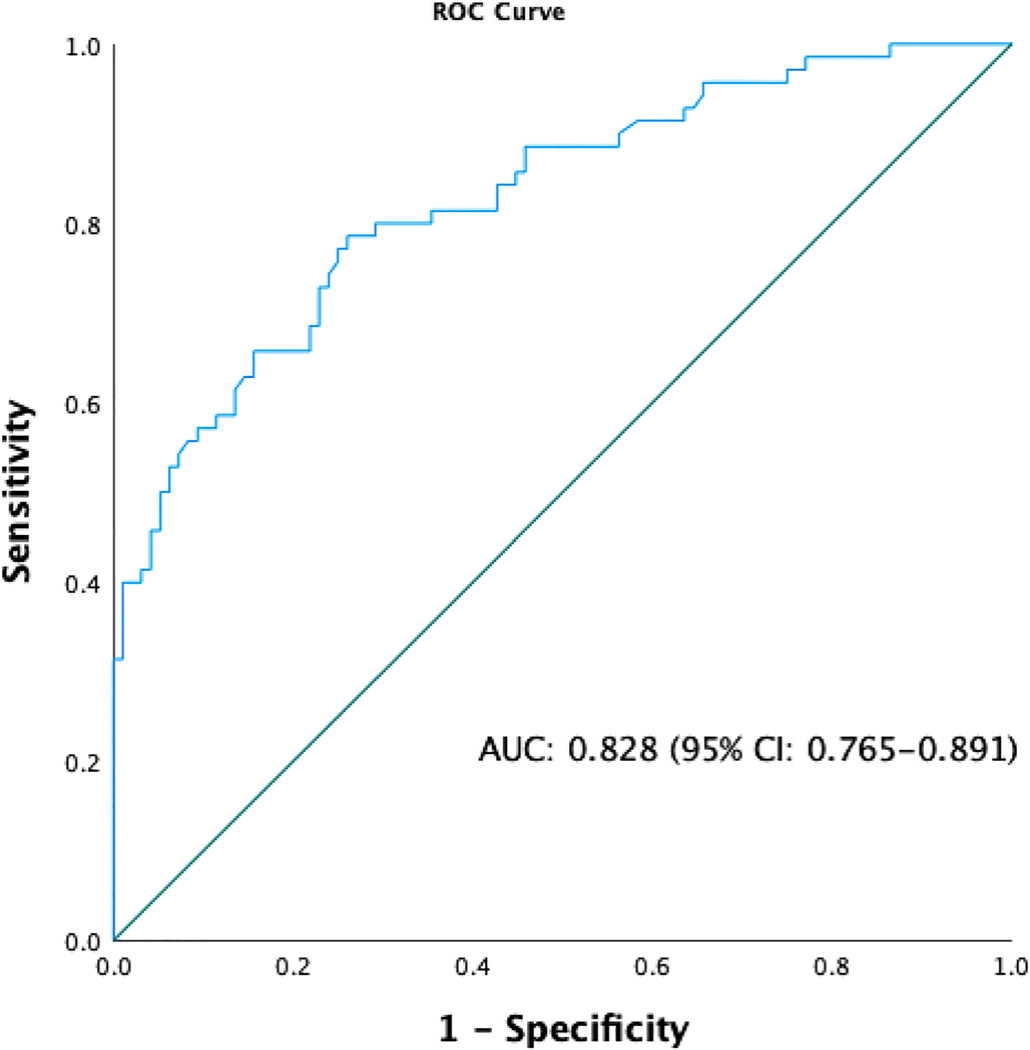
**ROC curve of the multivariable logistic regression model for perfusion impairment.** The ROC curve demonstrates the discriminative performance of the multivariable logistic regression model for identifying perfusion impairment. The model showed moderate-to-good discrimination, with an AUC of 0.828 (95% confidence interval: 0.765–0.891), indicating better overall predictive performance than the individual variables analyzed separately. The diagonal reference line represents no discriminative ability. Abbreviations**:** AUC, area under the curve; CI, confidence interval; ROC, receiver operating characteristic.

The ROC analysis of the multivariable logistic regression model demonstrated moderate-to-good discriminative ability, with an AUC of 0.828 (95% CI: 0.765–0.891), which was higher than that of the individual variables (Figure 5). Internal validation was performed using a bootstrap resampling procedure with 1,000 iterations to assess the stability of the model estimates. Bootstrap analysis confirmed the stability of the model, with high-grade AV block and age remaining statistically significant (bootstrap *P* ═ 0.002 and *P* ═ 0.001, respectively).

In multivariable logistic regression analysis (Table 5), age (OR 1.097, *P* ═ 0.001), heart rate (OR 0.910, *P* ═ 0.022), and high-grade AV block (OR 7.546, *P* ═ 0.002) remained independently associated with perfusion impairment. CKD, albumin levels, CRP, and troponin positivity did not demonstrate significant independent associations after adjustment.

In an additional analysis adjusting for baseline severity markers, including systolic blood pressure and creatinine, high-grade AV block remained independently associated with perfusion impairment (*P* ═ 0.002, OR = 8.6, CI = 5.2–14.1), consistent with the primary model.

The calibration of the final logistic regression model was assessed using the Hosmer–Lemeshow test, which indicated good model fit (χ^2^ ═ 7.686, df = 8, *P* ═ 0.465). The model also demonstrated moderate explanatory power (Nagelkerke R^2^ = 0.489). In addition, the calibration slope was 1.005, demonstrating good agreement between predicted and observed risks with no evidence of overfitting (Table 6).

**Table 4:** ROC analysis of age and heart rate in relation to perfusion impairment

**Variable**	**AUC (95% CI)**	**Cut-off**	**Sensitivity**	**Specificity**
Age (years)	0.719 (0.653–0.816)	≥ 72.5	0.829	0.490
Heart rate (bpm)	0.750 (0.672–0.829)	≤ 33.5	0.875	0.543

Abbreviations: AUC, area under the curve; bpm, beats per minute; CI, confidence interval; ROC, receiver operating characteristic.

**Table 5:** Factors associated with perfusion impairment: A multivariable logistic regression analysis

**Predictor**	**B**	**OR (95% CI)**	**Original *P***	**Bootstrap *P* (2-tailed)**
High-grade AV block	2.021	7.546 (2.119–26.868)	0.002	0.002
Age	0.093	1.097 (1.046–1.151)	0.001	0.001
Heart rate	--0.094	0.910 (0.840–0.987)	0.022	0.039
CRP	0.020	1.020 (0.999–1.042)	0.067	0.057
Albumin	--0.036	0.964 (0.863–1.078)	0.522	0.497
Troponin positivity	0.075	1.078 (0.421–2.763)	0.875	0.866
CKD	--0.256	0.774 (0.211–2.839)	0.579	0.560

Nagelkerke R square: 0.489; Hosmer–Lemeshow test: χ^2^ ═ 7.686, df = 8, *P* ═ 0.465; Internal validation: Results based on 1000 bootstra*p* samples. Abbreviations: AV, atrioventricular; B, regression coefficient; CI, confidence interval; CKD, chronic kidney disease; CRP, C-reactive protein; OR, odds ratio.

**Table 6:** Calibration analysis of the multivariable logistic regression model for perfusion impairment

**Variable**	**B**	**S.E.**	**Wald**	**Sig.**	**Exp(B)**	**95% CI lower**	**95% CI upper**
LOGITP	1.005	0.164	37.499	<0.001	2.731	1.980	3.766

Abbreviations: B, regression coefficient; CI, confidence interval; Exp(B), exponentiated regression coefficient; LOGITP, logit of predicted probabilities: S.E. standard error: Sig., significance.

**Table 7:** Distribution of perfusion impairment by exploratory risk score categories

**Risk category**	**No perfusion impairment**	**Perfusion impairment**	**Total patients**	**Prevalence (%)**
**Low risk**	84	28	112	25.0%
**Intermediate risk**	5	12	17	70.6%
**High risk**	7	30	37	81.1%
**Total**	**96**	**70**	**166**	**42.2%**

In an exploratory analysis, a simplified risk score was constructed based on the multivariable logistic regression results. Age ≥72.5 years and heart rate ≤33.5 bpm were each assigned 1 point, while the presence of high-grade AV block was assigned 3 points, reflecting its stronger association with perfusion impairment. The total score ranged from 0 to 5. Patients were categorized into three groups: low risk (0–1 points), intermediate risk (2–3 points), and high risk (4–5 points). The exploratory risk score effectively stratified the risk of perfusion impairment, with a significant stepwise increase in prevalence observed across the risk categories (25.0% in low-risk, 70.6% in intermediate-risk, and 81.1% in high-risk groups; Pearson chi-Square = 42.14, *P* < 0.001; Table 7). No additional performance metrics were calculated for this score due to the exploratory nature of the analysis.

As a supportive sensitivity analysis, 1:1 PSM was performed. Patients with and without perfusion impairment were matched based on age, heart rate, systolic blood pressure, and creatinine levels, resulting in a matched cohort of 90 patients (*n* ═ 45 per group). After matching, there were no significant differences in age (73.2 vs. 72.8 years, *P* ═ 0.845), heart rate (34.7 vs. 34.5 bpm, *P* ═ 0.812), or systolic blood pressure (108.1 vs. 109.2 mmHg, *P* ═ 0.775) between the groups. Standardized mean difference (SMD) analysis demonstrated improved covariate balance after matching across major baseline variables (Table 8). However, the prevalence of high-grade AV block remained significantly higher in the perfusion impairment group compared to the matched control group (62.2% vs. 26.7%, *P* ═ 0.001, SMD = 0.745) (Table 8).

**Table 8:** Comparison of clinical characteristics after propensity score matching between patients with and without perfusion impairment

**Variable**	**No perfusion impairment (*n* ═ 45)**	**Perfusion impairment (*n* ═ 45)**	***P* value**	**SMD**
Age (years)	72.8 ± 10.1	73.2 ± 9.5	0.845	**0.041**
Heart rate (bpm)	34.5 ± 3.9	34.7 ± 4.1	0.812	**0.050**
Systolic B*P* (mmHg)	109.2 ± 17.4	108.1 ± 18.2	0.775	**0.062**
Creatinine (mg/dL)	1.15 ± 0.42	1.19 ± 0.46	0.672	**0.091**
High-grade AV block, n (%)	12 (26.7%)	28 (62.2%)	0.001*	**0.745**

Data are presented as mean ± SD or n (%). SMD is used, with an SMD < 0.10 indicating negligible imbalance between groups for the matched covariates. Abbreviations: AV, atrioventricular; BP, blood pressure; n, number; SD, standard deviation; SMD, standardized mean difference.

## Discussion

In this study, we evaluated clinical factors associated with perfusion impairment in patients with symptomatic bradycardia undergoing permanent pacemaker implantation. Our findings indicate that advanced age, high-grade AV block, and lower baseline heart rate are independently associated with perfusion impairment. This study specifically focused on patients requiring permanent pacemaker implantation, representing a more severe and persistent form of symptomatic bradycardia, which should be considered when interpreting the findings. Within this selected population, hemodynamic compromise may be influenced by patient characteristics, with certain subgroups potentially at higher risk.

Bradycardia can lead to abrupt reductions in cardiac output by limiting ventricular filling and impairing stroke volume, ultimately resulting in decreased systemic perfusion and elevated filling pressures. These hemodynamic disturbances become particularly pronounced in patients with advanced conduction system disorders, predisposing them to symptoms such as fatigue, dyspnea, syncope, and, in severe cases, hemodynamic collapse. Recent guidelines and contemporary literature emphasize that patients with high-grade atrioventricular block or profoundly low heart rates constitute a high-risk subgroup in whom even modest bradycardia can precipitate significant circulatory compromise [1, 9–11]. Additionally, comorbidities such as CKD, anemia, pulmonary disease, and reduced cardiopulmonary reserve may further amplify susceptibility to perfusion impairment. Consistent with these observations, our study demonstrated that patients with perfusion impairment had significantly lower heart rates and a substantially higher prevalence of high-grade AV block, underscoring the central role of conduction severity in the development of hemodynamic instability.

Age is a major determinant of both bradycardia and perfusion impairment. Structural and degenerative changes in the cardiac conduction system, including fibrosis of the sinoatrial and AV nodes, reduce electrical conduction capacity, predisposing elderly patients to persistent bradycardia [5, 12, 13]. Consequently, older adults more frequently exhibit AV block, nodal rhythm, or other conduction disturbances, which increase the risk of systemic hypoperfusion. Our findings confirm that aging not only predisposes individuals to bradycardia but also independently elevates the risk of perfusion impairment, emphasizing the importance of vigilant monitoring in this population. Furthermore, studies have demonstrated that advanced conduction abnormalities, including high-grade AV block, can result in clinically significant hemodynamic compromise, particularly in post-procedural and high-risk populations [14].

The severity and persistence of bradycardia, along with the site of conduction disturbance (sinoatrial node, AV node, or His-Purkinje system), significantly influence clinical presentation [5, 6, 15]. Persistent bradycardia results in prolonged reductions in cardiac output, which may manifest as hypotension, fatigue, and alterations in mental status. In our cohort, altered mental status was observed more frequently than overt hypotension, suggesting that clinically relevant hypoperfusion may occur even in the absence of marked systemic hypotension, particularly in elderly patients with impaired autoregulatory mechanisms. This finding is consistent with previous studies that highlight the limitations of relying solely on systemic or biochemical markers to assess tissue perfusion [16]. Accordingly, patients with conduction system involvement, particularly those with high-grade AV block, are more likely to experience perfusion impairment. This aligns with previous research demonstrating an association between advanced AV conduction disturbances and hemodynamic compromise [14, 17]. Our analysis confirmed that high-grade AV block was associated with perfusion impairment, and this association remained significant after adjusting for potential confounders. PSM analysis indicated that the higher prevalence of high-grade AV block persisted after controlling for key baseline clinical variables, supporting the robustness of the observed association. While our PSM analysis improved balance across major clinical confounders, caution is warranted in interpreting the association between high-grade AV block and perfusion impairment. Given that high-grade AV block is the primary exposure of interest in this cohort, these findings illustrate a strong descriptive association rather than a definitive causal relationship. Therefore, the PSM findings should be viewed as supportive evidence of confounding control rather than conclusive.

In our cohort, a lower baseline heart rate was associated with perfusion impairment, suggesting a potential basis for risk stratification. An exploratory ROC-derived threshold of ≤33.5 bpm demonstrated high sensitivity but relatively low specificity. This indicates that while the threshold may be valuable for screening high-risk individuals, it should be interpreted cautiously in definitive clinical decision-making. Since confidence intervals for sensitivity and specificity were not calculated, these values should be considered descriptive and internally derived findings, requiring external validation before clinical application.

In addition to identifying independent factors associated with perfusion impairment, we constructed a simplified exploratory risk score to illustrate the potential clinical integration of these variables. This score combines age, heart rate, and high-grade AV block into an easily applicable framework that may facilitate preliminary bedside risk stratification.

However, this scoring system should be interpreted with caution, as it was derived from a single-center retrospective cohort and not subjected to formal validation or calibration analyses. Therefore, it should be considered hypothesis-generating, necessitating prospective studies to confirm its clinical utility.

These findings are consistent with prior evidence emphasizing the importance of early recognition of high-risk patients. Previous work underscores that baseline heart rate assessment via ECG is critical for the early recognition and management of symptomatic bradycardia [18]. Temporary or permanent pacing may rapidly restore systemic perfusion and prevent progression to hypotension, syncope, or cardiac arrest, particularly in high-risk patients [1, 14]. Moreover, long-term follow-u*P* data suggest that restoration of adequate heart rate and conduction can improve hemodynamic outcomes, even in complex cardiac populations, including transplant recipients [19]. This underscores the significance of early identification and intervention for high-risk patients.

Our study differs from previous work in several key aspects. Unlike studies focusing solely on acute kidney injury [20], we comprehensively evaluated systemic perfusion impairment using lactate levels, liver function tests, and changes in mental status. The inclusion of a broad spectrum of bradycardia—2:1 AV block, high-grade AV block, nodal rhythm, and atrial fibrillation with low ventricular response—allowed for a more detailed assessment of symptomatic bradycardia. These findings reinforce that high-grade AV block plays a predominant role in perfusion impairment, independent of bradycardia type. Furthermore, the integration of ECG-based evaluation and awareness of long-term outcomes can guide more precise risk stratification and clinical management.

Several limitations should be acknowledged. First, this was a single-center, retrospective study with a relatively modest sample size, which may limit the generalizability of the findings. Additionally, the inclusion of only patients who underwent permanent pacemaker implantation resulted in a selected cohort representing more severe and persistent forms of symptomatic bradycardia, which may further restrict the applicability of the results to the broader population.

Perfusion impairment was defined as a composite endpoint based on predefined objective clinical and biochemical criteria. Improvement after pacing was considered supportive evidence for bradycardia-related hypoperfusion but was not required for diagnosis. This approach aimed to minimize potential bias related to any inherent linkage between exposure and outcome. However, we acknowledge that including improvement after pacing in the clinical evaluation may still introduce a degree of linkage with heart rate and conduction severity. Since these components may differ in their clinical significance and underlying mechanisms, the heterogeneity of this endpoint may influence the interpretation of the results. The use of predefined and standardized criteria for endpoint assessment may partially mitigate this limitation.

Although the proportion of missing data was low, the use of complete-case analysis may have introduced a potential risk of bias. Furthermore, the ROC-derived cut-off values were not externally validated and should be interpreted with caution. Additionally, the relatively limited sample size may have affected model stability and generalizability. Since the multivariable model and the exploratory risk score were developed and assessed within the same dataset, the risk of overfitting cannot be completely excluded, necessitating external validation before clinical application.

Additional analyses including baseline severity markers yielded consistent results, supporting the robustness of the association between high-grade AV block and perfusion impairment.

Finally, long-term hemodynamic outcomes following pacemaker implantation were not systematically evaluated, which may limit further insights into risk modification strategies. Future multicenter, prospective studies are warranted to validate these findings, refine risk stratification, and establish practical clinical protocols for early intervention.

## Conclusion

In conclusion, among patients with persistent symptomatic bradycardia requiring permanent pacemaker implantation, advanced age, high-grade AV block, and lower heart rate were independently associated with systemic perfusion impairment. In our cohort, a heart rate below 33.5 bpm was associated with perfusion impairment; however, this threshold should be considered exploratory. Early recognition of patients at higher risk may facilitate timely clinical decision-making and appropriate monitoring strategies to prevent hemodynamic deterioration. These findings should be interpreted as exploratory and hypothesis-generating rather than for direct clinical application.

## Supplemental data

Graphical abstract


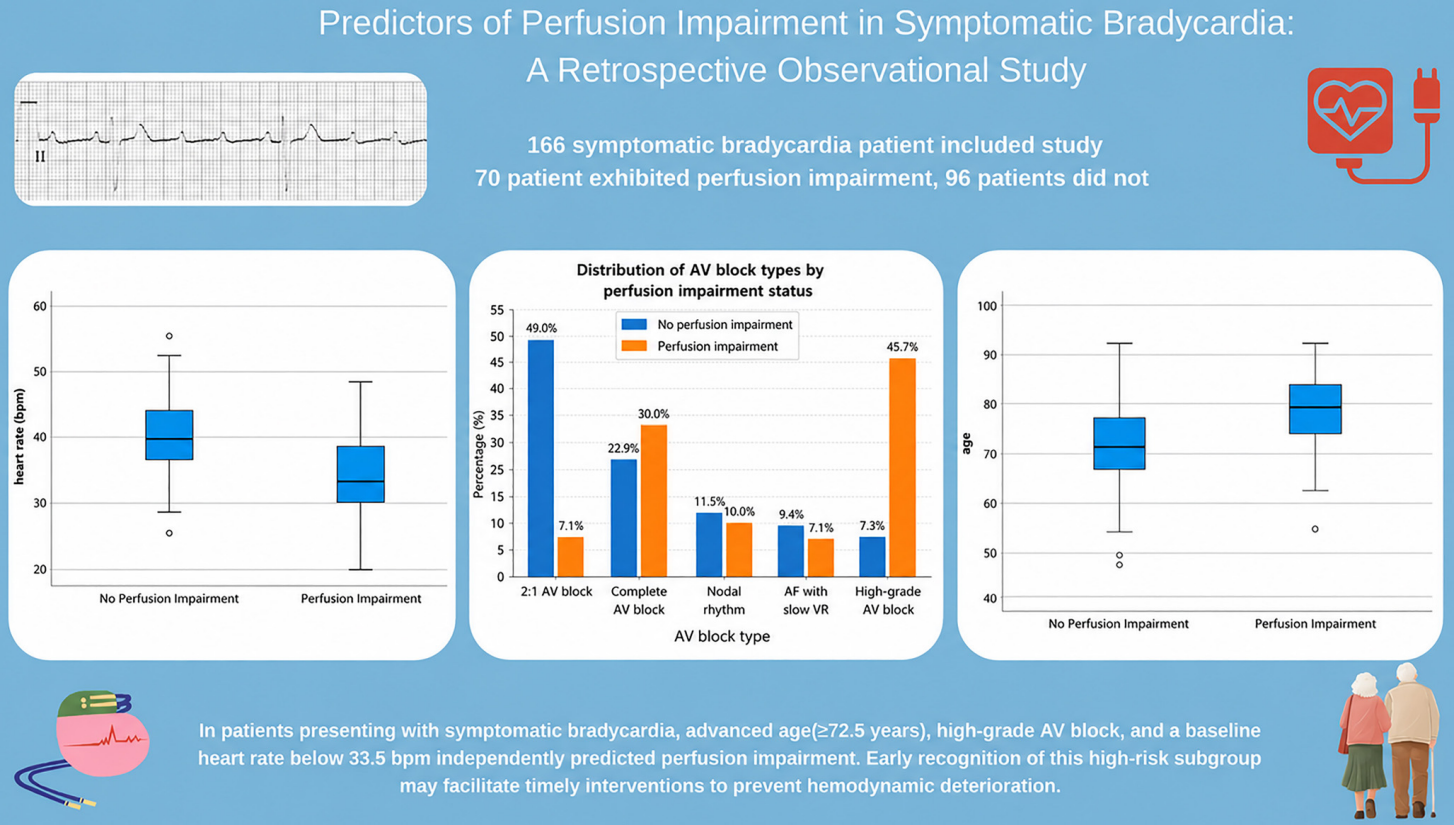


## Data Availability

The data sets created and/or analyzed during the current study are not publicly available because they contain patient information, but data supporting the findings of this study can be obtained from the corresponding author (RA) upon reasonable request.
